# Dorsal striatal astrocytes link dopamine to slow behavioral dynamics

**DOI:** 10.1101/2024.12.01.626240

**Published:** 2026-10-14

**Authors:** Iakovos Lazaridis, Emily Hueske, Gun Ahn, Wonchang Choi, Kojiro Hirokane, Sabrina Drammis, Ann M. Graybiel

## Abstract

Humans and other animals repeatedly pause from their goal-directed behavior by taking breaks. How rapid neuromodulatory signals help organize these daily engagement-disengagement cycles has remained unclear, as most studies have concentrated on in-task behavior. Here we added study of the breaks. We made simultaneous recordings of striatal dopamine release and astrocytic calcium activity as mice performed a decision-making task and took sporadic breaks. Dopamine release more strongly represented task events, whereas astrocytic calcium more strongly tracked disengagement during breaks and behavioral state cycles. Dopamine activity led to astrocytic activity, protracted adenosine signaling and dopamine depression. Blocking astrocytic calcium signals impaired engagement and rule-based learning. Astrocytes thus represent an essential aspect of the temporal organization of voluntary behavior, spanning the full engagement-disengagement cycles of daily life.

